# A High-Fidelity Texture Discretization Method for Polycrystalline Aggregates Considering Grain Size Distributions

**DOI:** 10.3390/ma19081501

**Published:** 2026-04-09

**Authors:** Hu Guo, Hui Huang, Jingrun Luo, Liling He, Xicheng Huang, Zhiming Hao

**Affiliations:** 1 Institute of Chemical Materials, China Academy of Engineering Physics, Mianyang 621999, China; 2 Institute of Systems Engineering, China Academy of Engineering Physics, Mianyang 621999, China; 3 China Academy of Engineering Physics, Mianyang 621999, China

**Keywords:** polycrystalline aggregate, grain size distribution (GSD), texture discretization, orientation distribution function (ODF), inverse transform sampling, total variation distance (TVD), binning strategy, polymer-bonded explosive (PBX)

## Abstract

**Highlights:**

- Robust texture discretization across diverse grain size and orientation distributions.
- Deterministic source samples are reconstructed from cumulative grain volumes.
- Discretized textures preserve probability mass and geometric periodicity.
- Discretization error (TVD) is reduced by one order of magnitude.
- Standard deviation of TVD is reduced by two orders of magnitude.

**Abstract:**

Accurate discretization of the orientation distribution function (ODF) is essential for reliable microstructural modeling of polycrystalline aggregates. This work proposes a novel texture discretization method that achieves high-fidelity ODF approximation even with a small number of orientations using only grain volume information. The core idea is to extend conventional inverse transform sampling by reconstructing the source samples before inversion. This reconstruction suppresses discretization errors induced by random sampling fluctuations and improves adaptability to non-uniform grain size distributions (GSDs). To preserve texture diversity under the same ODF, spatial shuffling and subsequent unscrambling of grain positions are introduced. The total variation distance (TVD) is adopted as a global metric to quantify discretization errors, and key influential factors are systematically analyzed, particularly the binning strategies. Error comparisons demonstrate that, within the typical range of grain numbers (10^2^–10^3^), the TVD of the proposed method is one order of magnitude lower than that of the conventional method, with its standard deviation two orders of magnitude smaller. The randomness and periodicity of discretized textures are further investigated, thereby elucidating the underlying mechanisms for the newly introduced advantages. This method provides a robust and efficient framework for texture modeling with consideration of GSDs.

## 1. Introduction

In both natural and industrial settings, a wide variety of materials are found in a polycrystalline state [1,2]. These polycrystalline aggregates are composed of numerous grains, each of which may possess its own morphology and crystallographic orientation [3]. When grains align preferentially along one or more specific directions, a crystallographic texture is formed [2,4]. This type of microstructural feature can exert a significant influence on the macroscopic physical, mechanical, and chemical properties of materials [5,6,7,8,9].

Accurate prediction of material performance requires representative microstructural models, which in turn depend on realistic grain morphologies and appropriately assigned crystallographic orientations [4,10]. If the number of modeled grains matches that of the actual material, the experimentally measured orientations can be incorporated directly. In practice, however, most models contain only 10^2^–10^3^ grains to balance accuracy with computational cost, far fewer than in the real microstructure [11,12,13,14,15,16,17]. Under these circumstances, the experimentally measured or theoretically derived orientation distribution function (ODF) must be discretized into a limited number of orientations. This approach—commonly referred to as texture discretization—seeks to retain the statistical characteristics of the original ODF while enabling tractable numerical computations.

Since the 1980s, a variety of texture discretization methods have been developed, which can broadly be classified into two categories. The first category comprises deterministic approaches. Their central idea is to partition the orientation space into discrete grids and reorganize them according to predefined criteria, leading some authors to refer to this group descriptively as the “CUT method” [18]. Kocks et al. [19] and Helming et al. [20] discretized textures by constructing nearly equi-distant (NED) grids in orientation space, with grain volumes determined iteratively by fitting recalculated pole figures to experimental data. Grains with weights below a specified threshold were subsequently removed. An alternative deterministic strategy is the Limited Orientation Distance (LOD) method of Tóth and Van Houtte [18]. It begins by identifying the orientation with the highest ODF intensity as a seed, and then aggregates the intensities of neighboring orientations whose disorientation from this seed is below a prescribed threshold. A further representative approach is the Integer Approximation (IA) method, originally developed by Leffers and Jensen [21] and later extended by Xie and Nakamachi [22] for finite element computations. In this method, Euler space is divided into uniformly spaced bins, and the number of grains assigned to each bin is estimated by multiplying the local orientation probability by a sufficiently large integer. A further refinement was proposed by Vuppala et al. [23], introducing optimization and perturbation steps to accommodate materials with non-uniform grain sizes. Although deterministic methods are straightforward to implement, they rely on threshold parameters and scaling constants. This dependence makes the resulting discrete textures sensitive to parameter selection and can artificially sharpen strong components while suppressing weaker ones.

The second category comprises probabilistic discretization approaches (STAT), which essentially follow the principle of inverse transform sampling [24]. In this framework, the original ODF is integrated to obtain its cumulative distribution function (CDF), whose inverse is then used to map uniformly distributed random numbers to orientations [18]. From a statistical standpoint, this procedure yields an unbiased estimate of the underlying ODF [18,25]. STAT offers two notable advantages. First, it requires no parameters aside from the number of samples, rendering it more robust than deterministic schemes. Second, it provides high computational efficiency, as each orientation is generated through a single inverse mapping without iterative or fitting procedures. Despite these merits, STAT exhibits two intrinsic limitations: (i) when the sample size is small, discretization errors become difficult to control, and (ii) due to its theoretical foundation, the method is inherently restricted to cases involving uniform grain sizes.

Recent efforts have sought to extend STAT and mitigate its practical limitations by integrating it with complementary modeling techniques. Melchior and Delannay [26] proposed an approach that allows the grain size distribution (GSD) and the ODF to be specified independently. The method first assigns orientations to elementary volumes by inverse transform sampling and then clusters them. While effective, this method requires extensive pairwise disorientation checks, which can become computationally demanding for large-scale microstructure models. Eisenlohr et al. [25] developed a hybrid sampling procedure (hybrid IA) that combines the deterministic IA method with STAT: IA is used when the number of required samples is small, whereas STAT is applied in the large-sample regime. This hybrid approach mitigates the artificial sharpening of the texture that arises when too few samples are drawn, and it has been adopted in the multiscale simulation framework DAMASK [27]. In addition, Biswas et al. [28] incorporated kernel density estimation into the hybrid IA formulation and examined the influence of bin counts. Bakhtiari et al. [29] combined inverse transform sampling with cellular automata to assign orientations to grains with non-uniform GSD through iterative exchanges of randomly selected grain orientations. Although these developments broaden the applicability of the probabilistic discretization approach, the two limitations discussed above have not yet been fundamentally addressed, motivating further methodological advances in texture discretization.

To avoid conceptual ambiguity, the texture discretization methods discussed above should be clearly distinguished from the class of “texture component” approaches. The latter aim to characterize crystallographic texture by identifying the orientation, intensity, and shape of the main texture components [30,31,32,33,34,35,36]. When discrete orientations are required, these discretization methods are also employed. For instance, Raabe and Roters [34] adopted an approach that essentially follows a probabilistic sampling strategy, and Kuhn et al. [33] employed a hybrid method integrating probabilistic and deterministic strategies within the framework proposed by Melchior and Delannay [26].

Motivated by the limitations of existing discretization strategies in terms of generality and fidelity, this work presents a structured extension of the conventional probabilistic discretization approach. A novel high-fidelity discretization method is proposed, featuring algorithmic simplicity and compatibility with various GSDs. The methodological framework, algorithmic implementation, and theoretical foundation are described in detail, followed by a comprehensive analysis of the characteristics and sources of discretization errors. Systematic error comparisons with the conventional discretization strategy under diverse GSDs are conducted to validate the advantages of the proposed method. In addition, discussions of randomness and periodicity in discrete textures are provided to elucidate the mechanisms governing the fidelity of discretization.

## 2. Methodological Framework

### 2.1. Overall Workflow

**Figure 1:**
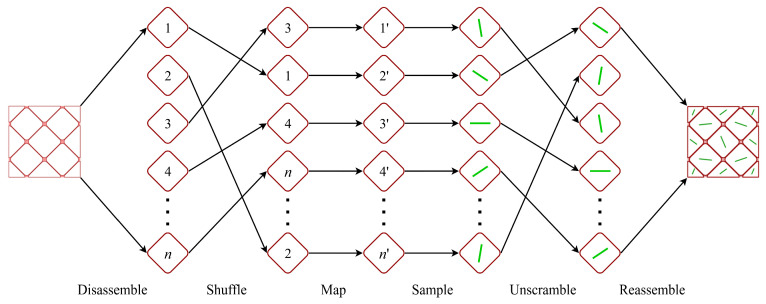
Fundamental process of the proposed texture discretization method (green lines indicate the spatial orientations of individual rhombic grains).

The ultimate objective of texture discretization is to assign spatial orientations to each grain within a microstructural model. This process transcends mere mathematical sampling; it embodies a microstructural construction strategy involving several steps beyond orientation sampling. The proposed texture discretization method consists of six fundamental steps: grain disassembly, order shuffling, index mapping, orientation sampling, order unscrambling, and grain reassembly. Figure 1 illustrates the basic workflow of the proposed method using a simplified microstructural model. The model consists of *n* uniformly distributed, equal-volume rhombic grains, each represented by a green line indicating its spatial orientation. To simplify the description of grain orientation, all grains are assumed to be disk-shaped, with atomic arrangements that exhibit mirror symmetry with respect to the principal plane and axial symmetry with respect to its normal [37]. Accordingly, the green line represents the normal to the principal plane of each grain. Although this idealized assumption is adopted for clarity, the proposed method is not limited to such grains and can also be applied to general grains without these symmetry characteristics.

Each of the aforementioned steps corresponds to a specific operation on the grains. Grain disassembly and grain reassembly are two complementary steps. They are designed to obtain and record the indices of individual grains within the model, thereby enabling accurate restoration of the original arrangement after the grain order has been shuffled. The initial indices $\left(1,2,3,\cdots ,n\right)$ may be generated according to specific ordering criteria, such as grain size. Order shuffling and order unscrambling likewise form a matching pair of steps. The purpose of order shuffling is to randomize the sequence of grains so that the shuffled order can be utilized for orientation sampling, thereby enhancing the diversity of the generated texture. It is essential to record the complete history of the shuffling operation to ensure accurate restoration during the unscrambling stage. To this end, an index mapping step is introduced to establish a one-to-one correspondence between the shuffled indices $\left(1^{\prime },2^{\prime },3^{\prime },\cdots ,n^{\prime }\right)$ and their original counterparts. Among these steps, orientation sampling represents the core of the entire process. It assigns a spatial orientation to each grain using an extended inverse transform sampling method, the mathematical implementation of which will be elaborated in the following sections.

### 2.2. Sampling Strategy

The conventional inverse transform sampling method is fundamentally based on the CDF, where the source samples used for the inverse transformation are required to follow a uniform random distribution [24,38]. Existing probabilistic texture discretization methods directly adopt this sampling strategy. However, they face two main limitations: the errors introduced by randomness are difficult to control, and the applicability is confined to cases with uniform grain volumes. To overcome these limitations, the present study extends the conventional inverse transform sampling approach by reformulating its underlying construction procedure. The source samples are determined from the grain volumes rather than from a random distribution. This eliminates the influence of randomness from source samples and, at the same time, broadens the applicability to various GSDs. In this paper, we do not distinguish between the size and the volume of a grain; both terms refer to the volume of an individual grain. For simplicity, the expressions *equal-volume grains*, *uniform grains*, and *uniform GSD* are used interchangeably to indicate that all grains have identical volumes.

**Figure 2:**
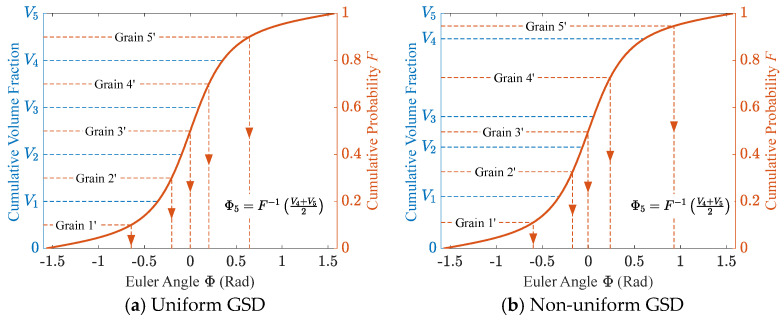
Schematic diagram of the extended inverse transform sampling method with (**a**) uniform and (**b**) non-uniform GSD.

To provide an intuitive illustration of the details of the extended sampling method, Figure 2 presents an example involving five grains (with both uniform and non-uniform volumes). The orientation angle of each grain is determined using the CDF of a modified March function. The March function, originally proposed by March A [39] and Dollase W A [37], is a concise ODF suitable for characterizing the orientation distribution of crystals possessing rod-like or disk-like axially symmetric structures. Luscher D. J. et al. [4] further proposed a modified March distribution function to account for the macroscopic volume variation in polymer-bonded explosives (PBXs) during the compaction process, expressed as:

(1) $$M\left(\varphi_{1},\Phi ,\varphi_{2}\right)=\lambda {\left(\sin^{2}\left(\Phi \right)+\lambda^{2}\cos^{2}\left(\Phi \right)\right)}^{-3/2}.$$

In this expression, a commonly used set of Euler angles (*φ*_1_, Φ, and *φ*_2_) following the Bunge convention [6] is employed, with Φ ranging from −π/2 to π/2. The parameter λ, defined as a real number between 0 and 1, could quantify the degree of orientation clustering of the grains. For consistency, Equation (1) is adopted as the original ODF in all subsequent sampling calculations, with λ fixed at 0.4. It should be noted that $\varphi_{1}$ and $\varphi_{2}$ do not appear in the expression, indicating that the function depends solely on Φ. The case involving coupling among the three Euler angles is beyond the scope of this study and will not be further discussed.

In Figure 2, $V_{i}\;(i=1,\ldots ,5)$ denotes the normalized cumulative volume fraction of the first i grains, $F\left(\Phi \right)$ represents the CDF of Equation (1), and $\Phi_{i}\;(i=1,\ldots ,5)$ corresponds to the orientation angle of the *i*-th grain obtained via inverse transformation. It is evident that this method essentially divides the probability axis based on cumulative volume fractions, followed by an inverse transformation. Therefore, apart from the cumulative volume information, it does not depend on any additional parameters. Moreover, it exhibits strong robustness across different GSDs.

### 2.3. Algorithm Implementation

**Figure 3:**
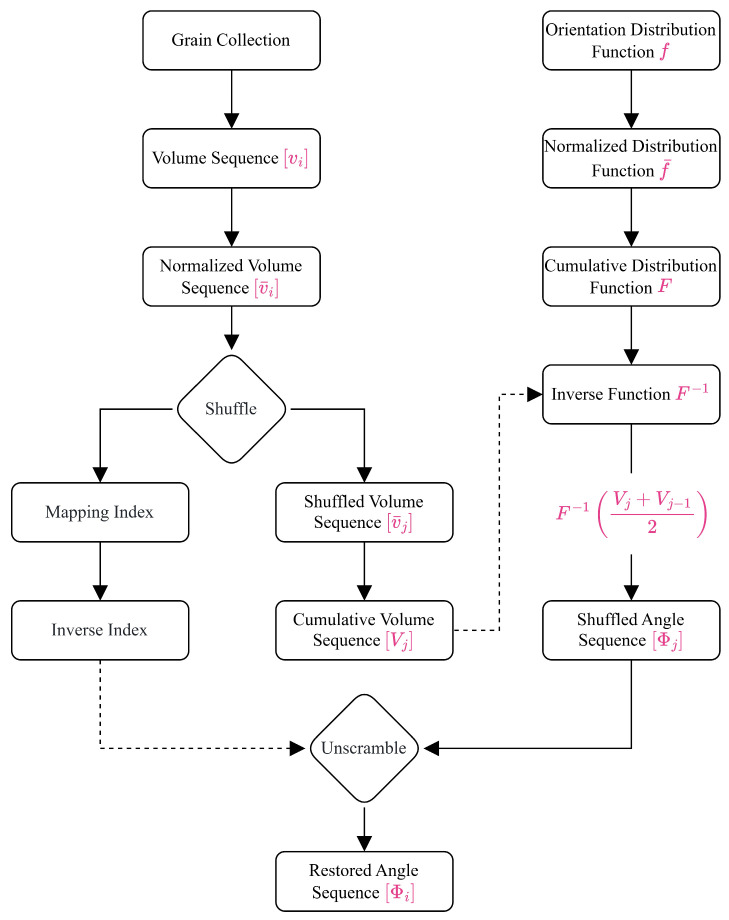
Algorithm flowchart of the proposed texture discretization method.

The texture discretization method proposed in this study involves the transformation of two objects—the grain volume sequence and the ODF—as well as data transmission between them. As illustrated in Figure 3, the method is implemented through two parallel operational chains. The left chain operates on grain data, whereas the right chain processes the ODF. Each chain consists of a sequence of operations. These two chains interact via data transmission and ultimately generate a sequence of orientation angles corresponding to each grain. In the figure, solid lines indicate the execution of specific operations, while dashed lines represent data flow across operations. To keep the flowchart more concise, the names of operations other than “shuffle” and “unscramble” are not labeled.

The left operational chain concerns the grain object and consists of five operations aimed at extracting and transforming grain volume data to provide the fundamental basis for subsequent orientation-angle sampling. The detailed operations include: (1) extracting the volume of each grain and constructing a volume sequence $\left[v_{i}\right]$ according to the grain indices; (2) normalizing the volume sequence to obtain a normalized volume sequence $\left[{\bar{v}}_{i}\right]$; (3) randomly shuffling the order of the grains to produce a new volume sequence $\left[{\bar{v}}_{j}\right]$ while recording the corresponding mapping index of grain identifiers, denoted by i→j; (4) inverting the mapping index to derive an inverse index, denoted by j→i; and (5) accumulating the shuffled volume sequence to generate the cumulative volume sequence $\left[V_{j}\right]$.

The right operational chain focuses on the ODF and likewise consists of five operations, which utilize the data generated from the first operational chain to perform the sampling of orientations. The detailed operations are as follows: (1) normalizing the original distribution function $f\left(\Phi \right)$ to obtain the normalized function $\bar{f}\left(\Phi \right)$; (2) integrating the normalized function to yield the CDF $F\left(\Phi \right)$; (3) solving for the inverse function $\Phi =F^{-1}\left(V\right)$; (4) computing the orientation angle of grain j using the inverse function $F^{-1}\left[\left(V_{j}+V_{j-1}\right)/2\right]$ to obtain the angle sequence $\left[\Phi_{j}\right]$ in shuffled state; and (5) employing the inverse index to unscramble the sequence $\left[\Phi_{j}\right]$, thereby obtaining the final angle sequence $\left[\Phi_{i}\right]$.

Through these two operational chains, each grain can be assigned a physically reasonable orientation angle. It should be noted that polycrystalline aggregates exhibit a wide variety of ODF types, some of which do not satisfy the normalization condition. In such cases, the normalization procedure in the right operational chain must be applied. In the absence of a closed-form expression for the CDF, the ODF should be normalized by numerical integration. Sampling is then performed using the inverse mapping interpolation method.

## 3. Correctness Proof

According to probability theory, a CDF is non-decreasing and right-continuous, with its range constrained to $\left[0,\;1\right]$. Let

(2) $$F\left(\Phi \right)=V,$$

where V and Φ are random variables, and F denotes the CDF of Φ. Then, the generalized inverse exists and can be expressed as:

(3) $$F^{-1}\left(V\right)=\inf\left\{\Phi \;\left|\;F\left(\Phi \right)\geq V\right.\right\},\;\left(0\leq V\leq 1\right).$$

Based on the fundamental relationship between the CDF and its generalized inverse, the following probabilistic identity holds:

(4) $$P\left(F^{-1}\left(V\right)\leq \Phi \right)=P\left(V\leq F\left(\Phi \right)\right).$$

Assume that V follows a uniform distribution on $\left[0,\;1\right]$, whose CDF is:

(5) $$H\left(V\right)=V\;V\in \left[0,\;1\right].$$

Combining Equations (4) and (5), we obtain:

(6) $$P\left[F^{-1}\left(V\right)\leq \Phi \right]=H\left[F\left(\Phi \right)\right]=F\left(\Phi \right).$$

It can be observed that a uniformly distributed variable (V) will, after the inverse transformation, follow the same probability distribution as the initial variable (Φ). This provides the theoretical foundation for the conventional inverse transform sampling method [24,38].

However, because the source samples used for the inverse transformation are drawn from a uniform population distribution, the conventional method applies only to microstructure models with equally weighted (or volumed) grains. Moreover, the inherent randomness of these samples can introduce nontrivial stochastic errors. When the sample size is small, such errors may significantly distort the sampling results, necessitating a substantially larger sample size to mitigate their effects. Therefore, if the source samples can be modified, the limitations of the conventional method may be overcome.

As discussed above, the modification of the source samples has two objectives: reducing random error and incorporating grain-volume information into the sample construction. We first examine the distributional characteristics of the source samples in inverse transform sampling. Here, the source samples refer to the samples obtained by mapping an arbitrarily distributed variable through its CDF.

For a random variable Φ of arbitrary distribution, if its cumulative probability $F\left(\Phi \right)$ is regarded as a variable, then the cumulative probability of this new variable can be written as $P\left[F\left(\Phi \right)\leq V\right]$. Combining this with Equation (4), we have:

(7) $$P\left[F\left(\Phi \right)\leq V\right]=P\left[\Phi \leq F^{-1}\left(V\right)\right]=F\left[F^{-1}\left(V\right)\right]=V.$$

It follows that, for any random variable, the value of its CDF is uniformly distributed on $\left[0,1\right]$. Thus, the probability axis, denoted by V, may be divided into numerous equidistant elementary intervals, each corresponding to a grain of equal volume. In this case, when one inverse-transform source sample is assigned to each elementary interval, the relation in Equation (6) remains valid, thus leading to a special case of the conventional inverse transform sampling method. In this setting, the random error of the source samples can be entirely eliminated. However, the method is still limited to systems with uniform grains.

To deal with non-uniform grains, an interval-combination strategy can be employed. Specifically, the elementary intervals are combined according to the volume fractions of the actual N grains, yielding N corresponding combined intervals. The interval associated with the *i*-th grain is denoted by $\left[V_{i-1},\;V_{i}\right.)$, where the subscript *i* represents the grain index. In particular, if no grain exists, $V_{0}=0$. Then, by applying the inverse transformation $F^{-1}\left(V\right)$ to the interval of an individual grain, the corresponding angular interval $\left[\Phi_{i-1},\;\Phi_{i}\right)$ can be obtained.

Assume that the probability mass associated with the angular interval $\left[\Phi_{i-1},\;\Phi_{i}\right)$ is $P_{i}$, then:

(8) $$P_{i}=P\left[F^{-1}\left(V_{i-1}\right)\leq F^{-1}\left(V\right)<F^{-1}\left(V_{i}\right)\right],$$

or equivalently,

(9) $$P_{i}=P\left[F^{-1}\left(V\right)<\Phi_{i}\right]-P\left[F^{-1}\left(V\right)\leq \Phi_{i-1}\right].$$

Since variable V remains uniformly distributed (though non-random), Equation (6) still holds. Substituting it into Equation (9) yields:

(10) $$P_{i}=F\left(\Phi_{i}\right)-F\left(\Phi_{i-1}\right).$$

Hence, the probability mass of each angular interval after the inverse transformation remains consistent with that of the corresponding interval in the original distribution. In other words, regardless of the form of the GSD, each grain’s angular interval preserves the statistical characteristics of the original distribution. Therefore, the correctness of the extended sampling method is proved.

The above derivation demonstrates that the core idea of the proposed extended sampling method lies in the strategic subdivision and combination of the probability axis, thereby discretizing the continuous variable domain into a set of independent subintervals. This operation not only extends the applicability of the inverse transform sampling method to non-uniform GSD but also effectively suppresses the stochastic errors resulting from source samples.

## 4. Error Analysis

### 4.1. Error Characteristics

**Figure 4:**
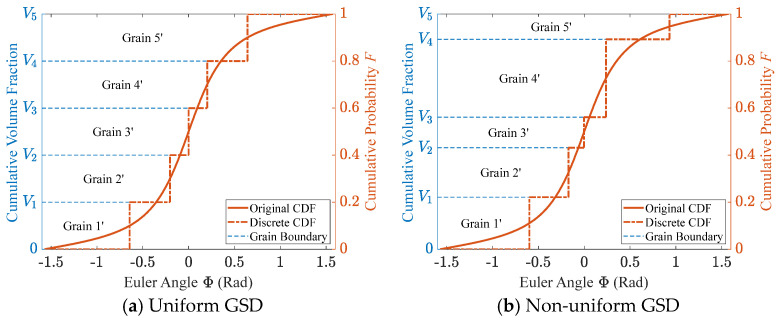
Comparison of the CDFs before and after discretization with (**a**) uniform and (**b**) non-uniform GSD corresponding to Figure 2.

Although the correctness of the extended inverse transform sampling method has been theoretically established, each grain in a microstructural model possesses only one orientation rather than being specified in the form of an angular interval. This inevitably introduces discretization errors, as in other texture discretization methods. If each grain’s orientation is assigned following the procedure in Figure 2, the resulting CDF takes the stepwise form shown in Figure 4. In this work, such a stepwise CDF is referred to as a discrete CDF, where the height of each step equals the volume fraction of the corresponding grain. Consequently, uniform grain sizes yield identical step heights (Figure 4a), whereas non-uniform grain sizes produce steps of varying heights (Figure 4b). However, step height alone is insufficient to accurately quantify the discretization error, since factors such as step width must also be considered. Therefore, suitable evaluation metrics are required for quantifying this discretization error. It should be noted that all “discrete” curves in this paper refer to curves after the discretization operation, without describing their geometric appearance.

### 4.2. Evaluation Metrics

The effectiveness of texture discretization may be evaluated qualitatively by comparing the pole figures generated from the discretized texture with those obtained experimentally. It may also be assessed indirectly through the ability of microstructure models based on the discretized texture to predict macroscopic properties [18,20,21]. However, for a more precise evaluation, the discrepancies between the original ODF and the discrete ODF are typically quantified. In previous studies, the methods used to measure these discrepancies can generally be classified into two categories: the L1 norm and the L2 norm. The former measures the absolute difference between the two density functions, providing an intuitive and concise form with a clear value range [0, 2], thereby offering stronger interpretability [25,28,36,40]. The latter measures the squared difference between the two density functions, which is smooth, differentiable, and optimization-friendly, but lacks a fixed value range and consequently exhibits weaker interpretability [23,26,30,33].

**Figure 5:**
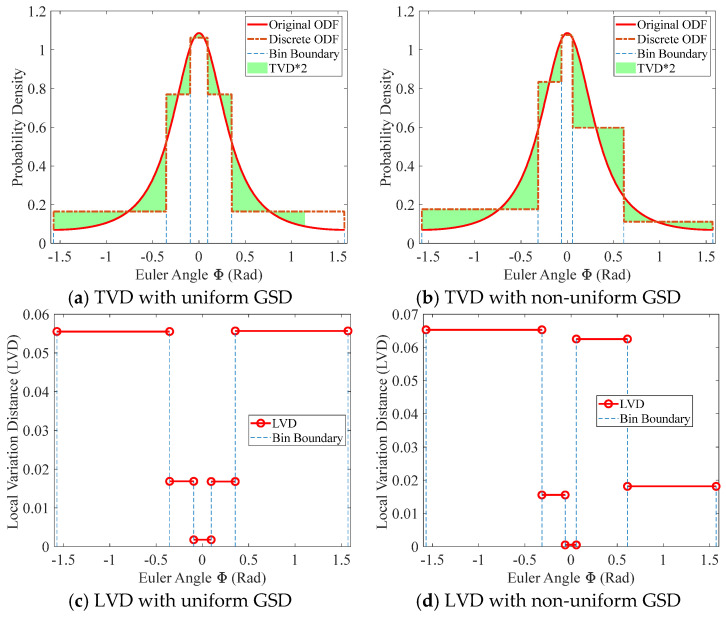
Metrics for evaluating discretization error during the discretization procedure corresponding to Figure 2. Top row: geometric interpretation of TVD for (**a**) uniform GSD and (**b**) non-uniform GSD. Bottom row: distributions of LVD for (**c**) uniform GSD and (**d**) non-uniform GSD.

In this work, we employ a new metric: the Total Variation Distance (TVD). This metric is similar to the L1 norm, but its range is [0, 1], making it a normalized measure that is more suitable for evaluating the discrepancy before and after discretization. Assuming that the original and discrete ODFs are two standard probability density functions denoted by f(Φ) and $\bar{f}\left(\Phi \right)$, respectively, their TVD can be expressed as [41]:

(11) $$\mathrm{TVD}\left(f,\bar{f}\right)=\frac{1}{2}\int \left|f\left(\Phi \right)-\bar{f}\left(\Phi \right)\right|d\Phi ,\;\Phi \in R,$$

where R denotes the union of the domains of the two functions. It is clear that TVD represents half of the total probability deviation between the two density functions. In the extreme case where the two functions are concentrated in non-overlapping regions, the TVD approaches 1. Conversely, when $\bar{f}\left(\Phi \right)$ approaches f(Φ), the TVD tends to 0. The top row of Figure 5 provides a geometric interpretation of the TVD, illustrating that it corresponds to half of the total area enclosed by the two distribution curves. In the figure, the green region represents the enclosed area, and the blue dashed lines (bin boundaries) partition the angular intervals corresponding to individual grains, consistent with the discretization procedure in Figure 2.

For the texture discretization process, TVD is a global measure and does not reflect the local distribution of errors. To analyze the local error characteristics, we further introduce the concept of Local Variation Distance (LVD). It can still be computed using Equation (11), except that the integration domain is restricted to the angular interval $R_{i}$ corresponding to an individual grain. LVD represents the error introduced by texture discretization at each grain; therefore, its value remains constant within each angular interval, as shown in the bottom row of Figure 5. This figure continues to correspond to the discretization procedure illustrated in Figure 2. The height of each red line indicates the error magnitude within its corresponding interval. Evidently, the LVD distributions under different GSDs vary significantly, highlighting the importance of local error analysis.

### 4.3. Influencing Factors

#### 4.3.1. Grain Count

From the definition of TVD (Equation (11)), the total discretization error depends not only on the step heights of the discretized probability curve but also on the widths of the corresponding angular intervals. For any given angular interval, its width remains constant. When the original ODF within this interval is discretized using grains of uniform volume, increasing the total number of grains reduces the step heights of the discretized ODF, thereby decreasing the error within that interval. Accordingly, for a polycrystalline aggregate composed of uniform grains, the TVD is expected to decrease as the grain count increases.

**Figure 6:**
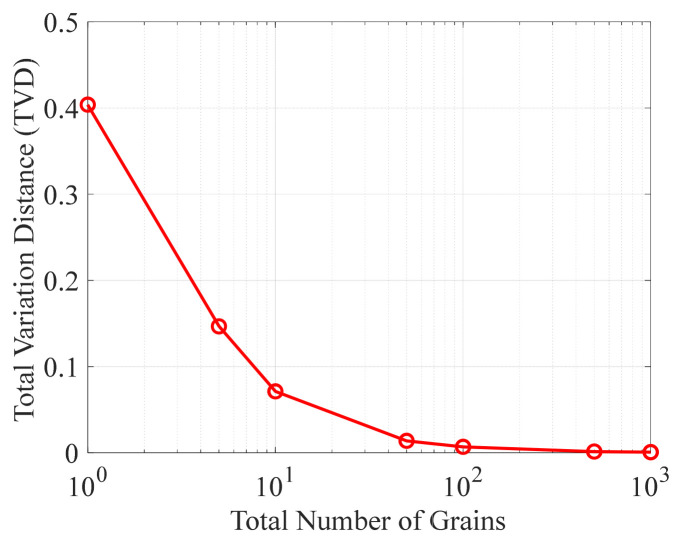
Relationship between TVD and the total number of uniform grains for the extended sampling method.

To further investigate the influence of grain count on TVD, the original ODF was discretized using the extended sampling method with uniform grains, and the resulting variation in TVD corresponding to grain count is shown in Figure 6. As observed from the figure, when the grain count is 1, the TVD is 0.404. As the grain count increases, the TVD decreases monotonically, and the rate of decrease gradually diminishes. When the total grain count reaches 100, the TVD drops to 0.0069, exhibiting a rapid decay trend. It should be noted that, because the horizontal axis is plotted on a logarithmic scale, the decreasing trend appears visually magnified. In summary, grain count is a key factor controlling the discretization error.

#### 4.3.2. Grain Ordering

From Figure 5a,c, it can be observed that placing grains of the same volume in different regions along the probability curve during discretization may lead to markedly different LVDs. In regions with lower probability density, the resulting LVD is larger. Conversely, in regions with higher probability density, the LVD is smaller and may even approach zero. This indicates that, for a polycrystalline aggregate with non-uniform grain volumes, different grain orderings may yield different discretization errors.

**Figure 7:**
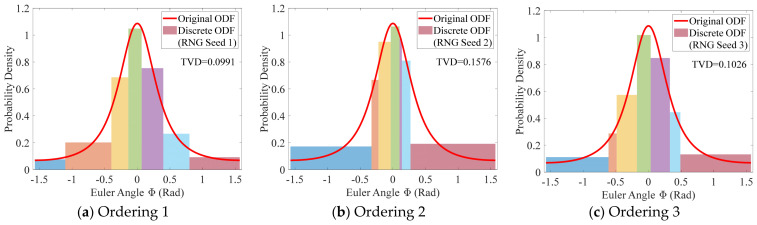
Discretization errors under different orderings of 7 non-uniform grains with extended sampling.

To investigate the influence of grain ordering, Figure 7 shuffles a sequence containing seven non-uniform grains using three different Random Number Generator (RNG) seeds and computes the discretization error for each shuffled case. The height of each rectangular bar represents the statistical probability density of the local interval, while the colors serve solely for visual distinction and carry no specific meaning. It can be observed that the error distributions differ significantly among the three cases. For example, in the case of ordering 2, the LVD is primarily concentrated on the first and last grains of the sequence, whereas this pattern is absent or much less pronounced in the other two cases. Further comparison shows that the TVDs are 0.099, 0.158, and 0.103, respectively, with the maximum relative deviation reaching 60%. The above analyses indicate that grain ordering is an important factor affecting the discretization error.

#### 4.3.3. Binning Strategy

In statistics, equal-interval binning and equal-frequency binning are commonly used data binning (grouping) strategies. The former divides the variable range into intervals of equal width, whereas the latter partitions the data such that each interval contains the same number of samples. In this study, all previous error estimations adopt the equal-frequency binning strategy. The rationale for this choice is that the extended inverse transform sampling method requires sequential, point-by-point sampling for each grain and ensures that the corresponding probability mass is correctly assigned. This point-wise sampling approach matches the logic of the equal-frequency data binning strategy with a frequency of one.

Typically, when the sample size is sufficiently large, the results obtained by equal-interval binning gradually converge to the theoretical distribution. However, under conditions of limited sample size, this method is highly sensitive to the number of bins. Too few bins lead to overly coarse statistics and obscure structural details of the distribution. In contrast, too many bins introduce substantial noise, making the overall distribution pattern difficult to interpret. Consequently, when the sample size is insufficient, equal-interval binning often results in considerable estimation errors.

**Figure 8:**
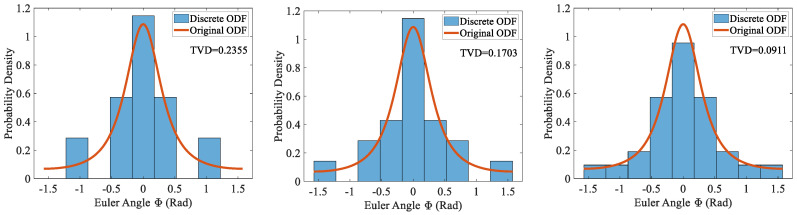

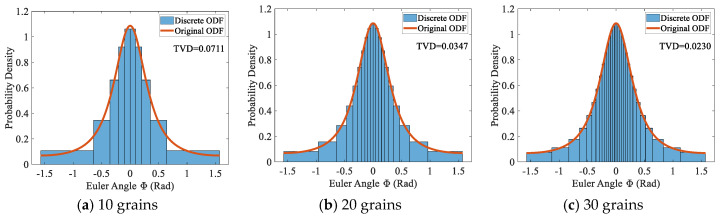
Statistical comparison of discretization errors for uniform grains with extended sampling: equal-interval (**top**) vs. equal-frequency (**bottom**).

Figure 8 presents the discretization errors produced by the extended sampling method under the uniform grain condition, with total grain counts of 10, 20, and 30. The upper row shows results based on equal-interval binning (uniformly divided into nine bins). When the total number of grains is only 10, the samples are extremely sparse across bins, and some bins even exhibit zero frequencies. This causes probability mass to be incorrectly allocated, leading to significant estimation bias. As the number of grains increases, zero-frequency bins gradually disappear, and the TVD decreases accordingly. For comparison, the lower row displays the results obtained using equal-frequency binning with unit frequency. It is evident that the TVD under this binning strategy is substantially lower than that of equal-interval binning, and no zero-frequency bins occur. Thus, under limited sample conditions, equal-frequency binning demonstrates notably superior estimation stability.

**Figure 9:**
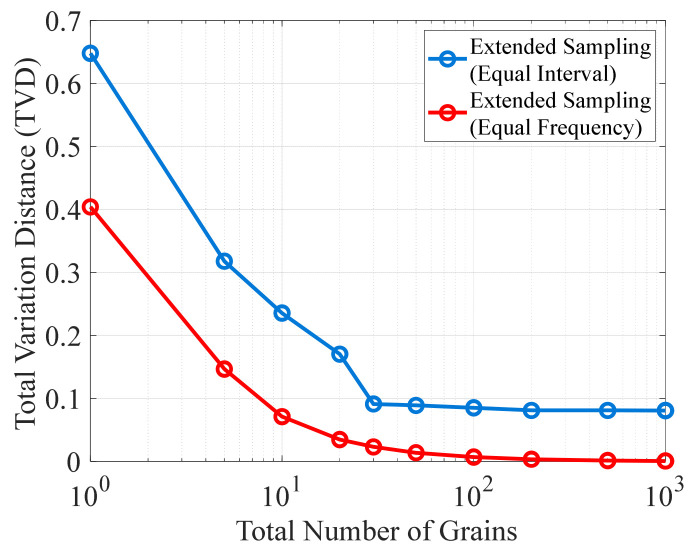
Comparison of TVD curves from extended sampling for uniform grains: equal-interval binning (blue) vs. equal-frequency binning (red).

Figure 8 shows that as the grain count increases, the TVDs obtained from both binning strategies exhibit decreasing trends, and the difference between them gradually diminishes. To further investigate this phenomenon, Figure 9 presents the TVD curves as functions of grain count for the two binning strategies, with the grain count ranging from 10^0^ to 10^3^. As the grain count increases, the TVD curve under the equal-frequency binning decreases monotonically, with the rate of decrease gradually slowing down. When the grain count reaches 1000, its TVD can be reduced to 0.0007. In contrast, although the TVD under the equal-interval binning also decreases monotonically, the change in its decreasing rate is more complex. For grain counts below 30, the TVD exhibits a relatively large overall rate of decrease, but with noticeable fluctuations. Thereafter, the rate of decrease drops sharply and becomes nearly constant. When the grain count increases to 200, the TVD obtained with equal-interval binning is approximately 0.0809, and further increases in grain count lead to little additional change. Combined with Figure 8, this indicates that the disappearance of zero-frequency bins may be the primary cause of the abrupt shift in the decreasing rate.

As shown in Figure 5a,b, TVD is directly related to the magnitude of probability jumps between adjacent bins. For equal-interval binning, because the number of bins is fixed, the probability jumps gradually stabilize as the grain count increases. In contrast, under equal-frequency binning, the number of bins increases with the grain count, causing the probability jumps to continuously decrease and approach zero. These differences in the patterns of probability-jump variation are precisely the fundamental reason why the two binning strategies exhibit different phenomena in error estimation.

### 4.4. Comparison with Conventional Method

#### 4.4.1. Determining the Binning Strategies

To compare the discretization errors of the conventional inverse transform sampling method and the proposed method, it is essential to first determine a binning strategy for the conventional method that minimizes them. Consistent with the previous discussion, both equal-interval and equal-frequency strategies are considered as binning options. For the conventional sampling method, the subsequent analysis is based on the following two settings: (1) the grain sizes are assumed to be uniform; and (2) different RNG seeds are used to represent the random nature of the sampling process.

**Figure 10:**
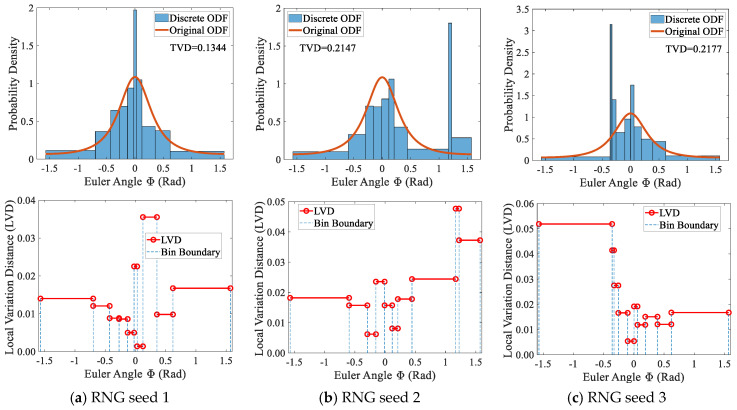
Equal-frequency statistical discretization errors for uniform grains with conventional sampling: TVD (**top**) and LVD (**bottom**).

When the discretization error of the conventional sampling method is evaluated using equal-frequency binning, three error distributions are obtained, as shown in Figure 10. These correspond to three sampling processes that utilize different RNG seeds, with each discrete sampling involving 10 uniform grains. The upper row of subfigures compares the original ODF with the discretized ODF and presents the corresponding TVD values. The results show that the TVD varies substantially across the three sampling processes, with the maximum relative deviation reaching 62%, indicating that sampling randomness can induce significant error fluctuations under identical discretization conditions. The lower row of subfigures displays the corresponding LVD distributions. Observations reveal that the LVD exhibits random variations among grains and may produce large local errors in individual grains, which partly explains the pronounced differences observed in the TVD.

**Figure 11:**
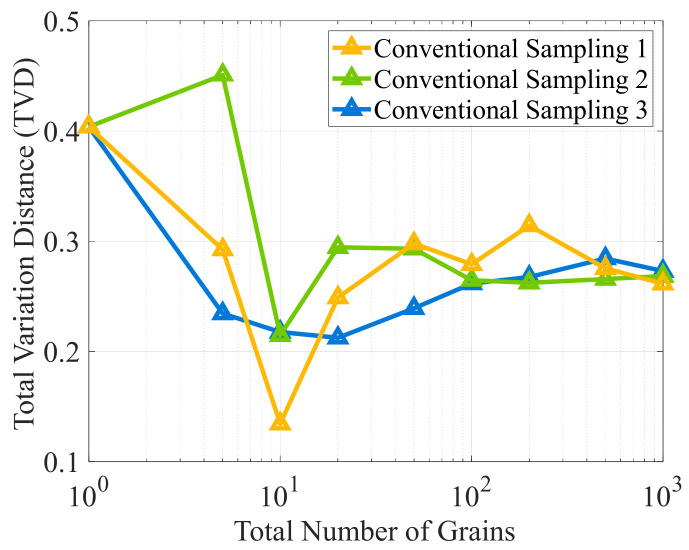
Equal-frequency statistical discretization errors for uniform grains with conventional sampling: TVD vs. grain count.

Figure 11 illustrates the trends of TVDs with the total number of uniform grains when employing an equal-frequency binning strategy to evaluate the errors associated with the conventional sampling method. The three curves correspond to different RNG seeds and characterize the sampling variations caused by randomness under the same discretization conditions. As the number of grains increases, the TVDs do not decrease monotonically; rather, they fluctuate initially and then gradually converge. When the grain count reaches 1000, the results from all three sampling processes stabilize at approximately 0.27. This level of error is relatively high and insufficient to accurately represent the characteristics of the original ODF, making it generally unacceptable in practical applications.

These findings indicate that the equal-frequency binning strategy is not suitable for the conventional sampling method, whereas the equal-interval binning strategy is more appropriate. In summary, the conventional sampling method should employ equal-interval binning with the number of bins adjusted according to the total number of grains (see Appendix A for guidelines), while the extended sampling method should adopt equal-frequency binning with one sample assigned to each bin.

#### 4.4.2. Comparative Analysis of TVDs

**Figure 12:**
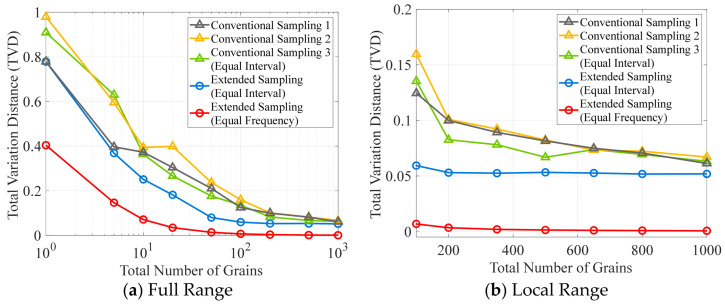
Comparison of error curves obtained using different sampling methods and binning strategies: TVD vs. grain count.

For ease of comparison, Figure 12 presents the variation in TVDs with the total number of grains for the two sampling methods. In the figure, the three curves marked with triangles represent three repeated discretization processes of the conventional method using different RNG seeds, whereas the curves marked with circles correspond to the extended sampling method. For the extended sampling method, in addition to the result estimated using equal-frequency binning (red), the result obtained using equal-interval binning (blue) is also provided. For all curves based on equal-interval binning, the number of bins is fixed at 15. It can be observed that, among all error curves, the TVD estimated for the extended sampling method using equal-frequency binning is the smallest. When the grain count equals 10, its TVD is already less than 0.1; when the grain count increases to 1000, its TVD decreases to 0.0007, significantly lower than the other four error curves. For these four curves using equal-interval binning, the TVDs gradually decrease with the increasing number of grains, yet the error levels remain relatively high. Specifically, at 1000 grains, the TVD values for the conventional method range from 0.0613 to 0.0670, whereas the TVD for the extended method is distinctly lower at 0.0518. This demonstrates that, under either equal-interval or equal-frequency binning, the extended sampling method exhibits lower discretization error than the conventional sampling method.

**Figure 13:**
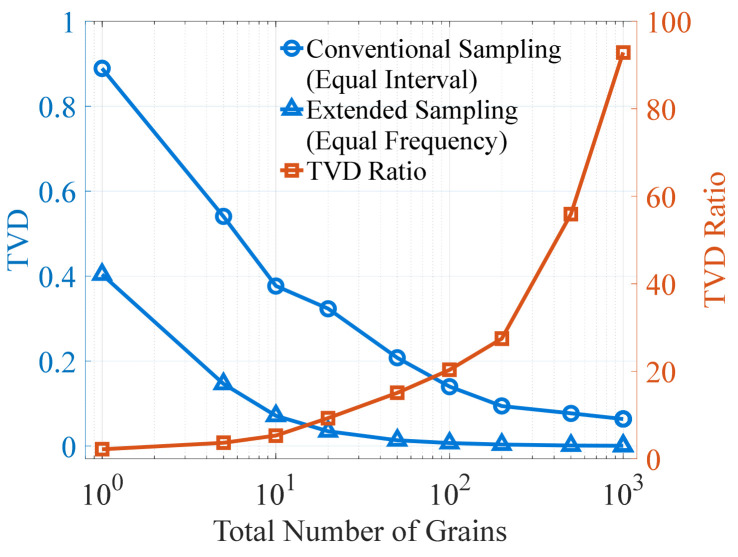
Variation in the TVD Ratio (Conventional vs. Extended Sampling) with Respect to Grain Count.

To evaluate the difference in the errors generated by the two sampling methods, the ratio of their TVDs is plotted against the total number of grains, as shown in Figure 13. The TVDs for the conventional method are obtained as the average over three repeated discretization processes, whereas the TVDs for the extended method correspond to the equal-frequency estimation. The data demonstrate a continuous increase in the TVD ratio with an increasing number of grains, and indicate a trend of further growth even beyond the upper limit of the grain count shown in the figure. Specifically, within the grain-count range commonly used in microstructure modeling (10^2^ to 10^3^), the TVD of the conventional method is approximately 20 to 93 times larger than that of the extended method. This indicates that the discretization error of the extended sampling method is roughly one order of magnitude lower than that of the conventional method. Consequently, applying the extended sampling method for discretization is expected to significantly enhance texture fidelity.

## 5. Validation Under Non-Uniform Grains

As demonstrated earlier through a comparison of discretization errors, the proposed texture discretization method can improve texture fidelity under uniform grain size conditions. However, when the grain size is non-uniform, discretization errors are also influenced by additional factors such as grain ordering (see Section 4.3.2). Therefore, this section applies the proposed method to various GSDs in order to verify its adaptability when the grains are non-uniform.

### 5.1. Grain Generation

Generating grains that conform to a specified GSD is a prerequisite for this validation study. Polycrystalline aggregate materials encompass a wide range of types, and their GSDs are highly diverse; even within the same class of materials, different distribution forms may arise. A typical example is polymer-bonded explosives (PBXs), which consist of explosive grains embedded in a polymer matrix. Their GSDs often exhibit unimodal, bimodal, or trimodal characteristics due to variations in formulation and processing [11,42,43,44,45,46]. This paper employs these three representative GSD types to investigate the adaptability of the proposed texture discretization method.

**Figure 14:**
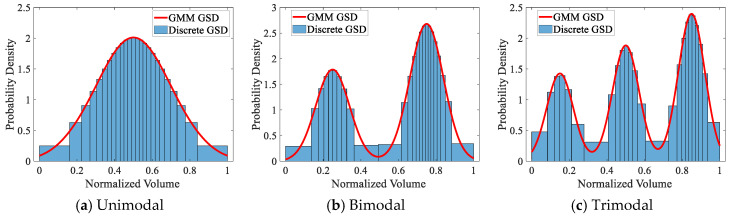
Generation of 25 grains using the extended sampling method for three types of GSDs.

To facilitate implementation, the Gaussian mixture model (GMM) is adopted to construct the distribution functions. The GMM represents a probability distribution as a weighted sum of multiple Gaussian components [47]. Owing to its flexibility and strong descriptive capability, it has been widely employed in image segmentation, machine learning, clustering analysis, and speech recognition [48,49,50]. The three continuous red curves in Figure 14 are constructed using GMM, representing unimodal, bimodal, and trimodal GSDs, respectively. During construction, the model parameters were carefully adjusted to ensure that the peaks of each curve are well separated.

Given a known GSD, accurately discretizing a specified number of grains is a nontrivial task. To achieve this, the fundamental idea of the extended sampling method proposed in this paper is adopted. Unlike texture discretization, grain generation involves discretizing the entire volume of the aggregate material, which is hereafter referred to as “volume discretization”. In this context, the horizontal axis in Figure 2 is changed to represent the normalized grain volume, while the vertical axis represents the probability density derived from the grain count. It is worth noting that some studies normalize the vertical axis by grain volume fraction when performing size statistics. Although this introduces additional complexity, it does not fundamentally affect the validation of the texture discretization method.

Furthermore, to highlight the advantages of the proposed method when the grain count is small and to facilitate visualization of the discretization effect, the total number of grains is fixed at 25. Figure 14 illustrates the GSD after volume discretization, where each bin corresponds to one grain and the bin height indicates the probability density of grain occurrence within each interval. Analogous to texture discretization, this histogram is referred to as the “discretized GSD”. A comparison between the original and discretized GSDs reveals a high level of agreement across all three distribution types, indicating that the error introduced by the volume discretization process is minimal.

As in texture discretization, the volume discretization error can also be quantified using TVD, which contributes to the modeling error associated with microstructural representations. However, this study focuses solely on texture discretization, whose discretization error is independent of the volume discretization error. Therefore, only the grain generation method is introduced here, without further analysis of the corresponding errors.

### 5.2. Texture Discretization Results

#### 5.2.1. Ordered Grain Arrangement

**Figure 15:**
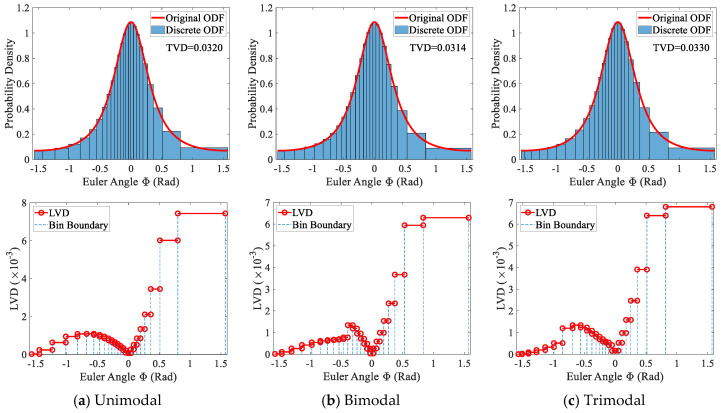
Texture discretization results of ordered grains with three GSDs: discrete ODF (**top**) and LVD (**bottom**).

The proposed texture discretization method consists of six steps (see Figure 1). If the step involving the shuffling of grain arrangements is omitted, the sampling step can be performed according to a specific order. As described in the previous section, the generated grains are sorted in ascending order of volume, thereby allowing the extended inverse transform sampling to follow this volume-based order. Using this order, Figure 15 presents the discretization results for different types of GSDs. The top subgraphs present the statistical distributions of the sampled orientations using equal-frequency binning with a frequency of 1. It can be observed that, for all three types of GSDs, the original ODF and the discretized ODF are in good agreement. The computed TVDs for the unimodal, bimodal, and trimodal GSDs are 0.0320, 0.0314, and 0.0330, respectively, with the maximum relative deviation being approximately 5%. These results indicate that the GSD type has a measurable effect on the discretization error.

Further comparison of the LVD distributions for the three GSD cases (see the bottom subgraphs of Figure 15) shows that their overall trends are largely similar. Because the grains are arranged from small to large volume, the last grain in the sequence has the largest volume. At the same time, the probability density of the original ODF is lowest near the boundaries of the orientation interval. As a result, the discretization error tends to accumulate in a small number of grains that possess both large volumes and large Euler angles. In addition, the LVD values tend to approach zero when the Euler angle approaches the lower bound of the interval or becomes zero. When the angle is near the lower bound, this occurs because the grain volume becomes extremely small. When the angle is zero, this behavior results from an extremum in the original ODF at that location, which produces the maximum slope of the CDF curve.

#### 5.2.2. Disordered Grain Arrangement

**Figure 16:**
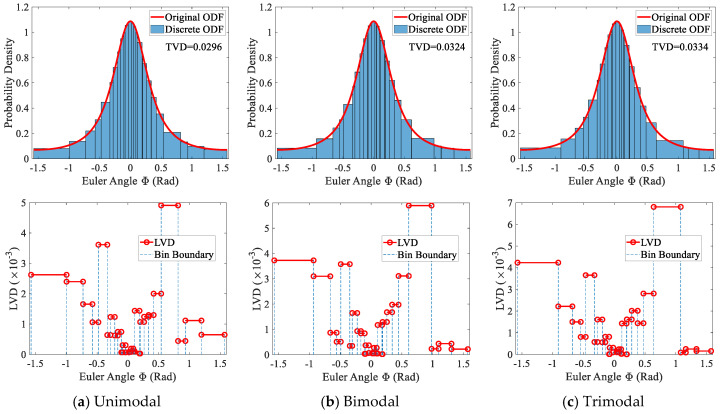
Texture discretization results of disordered grains with three GSDs: discrete ODF (**top**) and LVD (**bottom**).

The purpose of introducing the step that shuffles the grain order is not only to reflect the spatial randomness of texture but also to distribute the discretization error more uniformly among individual grains. Figure 16 presents the sampling results for the three GSD cases after randomly shuffling the grain order, where the same RNG seed is used for all cases. The top subgraphs compare the original ODF with the discretized ODF using equal-frequency binning with a frequency of 1. For the unimodal, bimodal, and trimodal GSDs, a high level of agreement between the original and discretized ODFs is still maintained, with TVDs of 0.0296, 0.0324, and 0.0334, respectively. Relative to the case where grains are sorted by volume (see Figure 15), the TVDs change by −8%, 3%, and 1%. Thus, shuffling the grain order can either increase or decrease the total discretization error. The uncertainty comes from the fact that the grain order controls when each grain is sampled. After shuffling, this timing changes, causing the LVD of a grain to shift either upward or downward, and thus affecting the overall TVD.

Although the overall TVD may vary unpredictably, the bottom subgraphs of Figure 16 clearly show that shuffling the grain order leads to a more uniform distribution of LVD. For all three GSD types, the LVD becomes more dispersed after shuffling rather than being concentrated in only a few large grains. According to Equation (11), the LVD of an individual grain is primarily influenced by its volume fraction and the local probability density of the ODF used in inverse transform sampling. Smaller grains or grains sampled in regions of higher probability density tend to receive lower LVD. After shuffling, large grains have a higher likelihood of moving away from low-probability regions, thereby preventing excessive accumulation of LVD. Consequently, shuffling helps redistribute the discretization error more evenly across grains, even though the TVD itself may fluctuate. It is also worth noting that, because the same RNG seed is used for all three GSD cases, their LVD distributions show similar fine-scale features, which is an expected consequence of the same underlying random process.

#### 5.2.3. Fidelity Analysis

During the texture discretization process, fidelity characterizes the ability of the discretized texture to reproduce the original texture. However, fidelity is usually not a single scalar, but rather a relative concept. Therefore, in this work, the fidelity of texture discretization is quantitatively evaluated from two aspects, namely “accuracy” and “precision”. Accuracy is measured by the magnitude of TVD, with a smaller TVD indicating higher accuracy. Precision is evaluated by the standard deviation (SD) of TVD obtained from repeated discretizations under identical conditions, where a smaller SD indicates higher precision.

**Figure 17:**
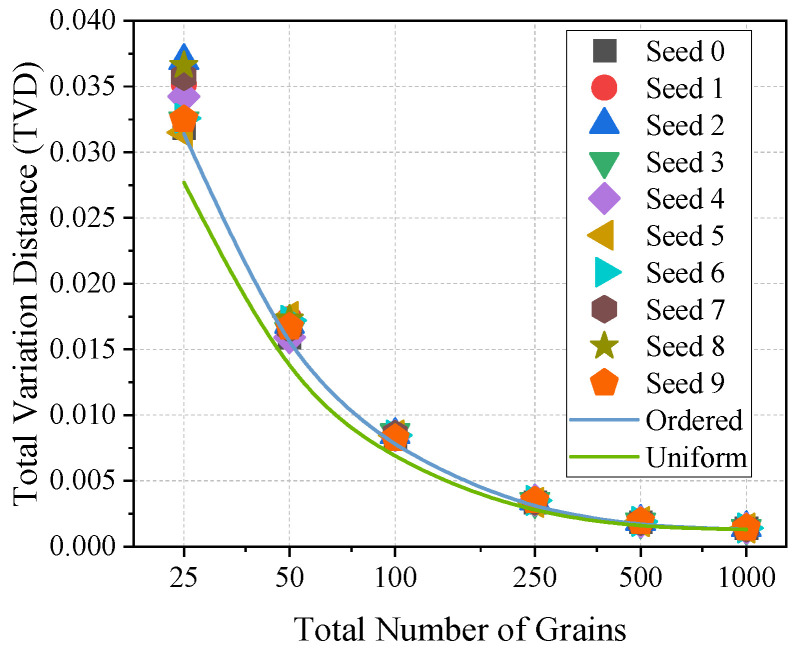
Variation in TVD with total grain count for the proposed texture discretization method. Shown are scatter results from randomized grain order, together with the ordered-grain case (blue line) under the bimodal GSD and a uniform-GSD reference (green line).

For the texture discretization method proposed in this work, repeated discretizations are realized by shuffling the grain sequence with different RNG seeds. The scatter points in Figure 17 show the TVD values corresponding to 10 repeated discretizations, where the RNG seeds range from 0 to 9, while all the GSDs follow the bimodal distribution depicted in Figure 14b. It can be seen that, for a fixed total grain number, the dispersion of TVD decreases as the grain count increases: when the grain count is 25, the maximum relative deviation of TVD is 18%; when the grain count increases to 1000, this deviation decreases to 10%. In addition, the blue reference line in Figure 17 corresponds to the case where the grains are arranged in order. A comparison shows that the TVD values for randomized grain orders are generally larger, suggesting that shuffling the grain sequence tends to amplify the overall error. It should be noted that the number of repeated discretizations is not necessarily optimal in a strict statistical sense; however, for the conditions considered in this study, it is sufficient to capture the main statistical characteristics of TVD. In the subsequent repeated discretizations of the conventional method, the same number of repetitions and the same RNG seed settings are adopted to ensure the comparability of the two discretization methods.

In the absence of a unified benchmark, it is difficult to directly assess the magnitude of TVD. Therefore, Figure 17 additionally presents the green TVD curve obtained under uniform GSD conditions as another reference. All TVD scatter points lie above this curve, indicating that the overall discretization error is minimized under a uniform GSD. By averaging the TVD values over 10 repeated discretizations and comparing them with the TVD corresponding to the uniform GSD, it is found that their difference decreases as the grain count increases. Specifically, the relative deviation is 23% when the grain count is 25, whereas it decreases to 8% as the grain count increases to 1000. When this proportional relationship is combined with the results in Figure 13, with the TVD of the conventional method under a uniform GSD retained as the baseline, a simple conversion can be used to further estimate the error of the proposed method under a non-uniform GSD. The results show that, within the range commonly used in microstructure models (10^2^ to 10^3^), the TVD of the conventional method is about 16 to 86 times that of the proposed method. This indicates that even under non-uniform GSD conditions, the error of the proposed approach is still lower by one order of magnitude.

**Figure 18:**
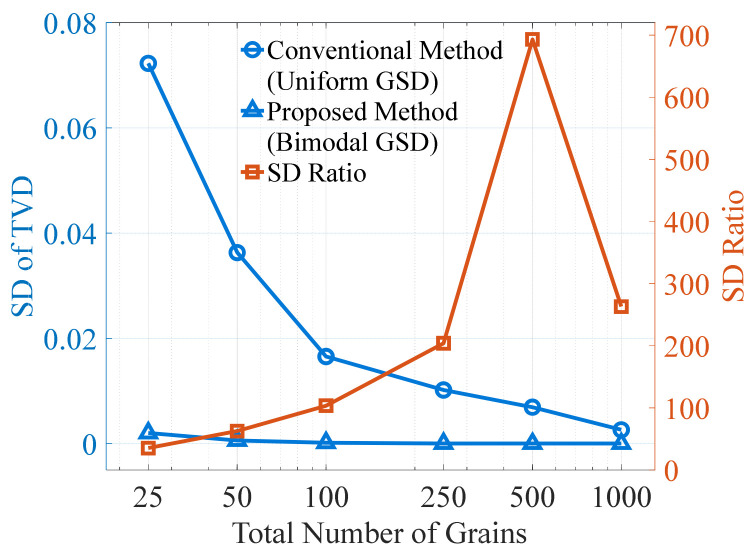
TVD standard deviations obtained from 10 repeated discretizations using the conventional (uniform GSD) and proposed (bimodal GSD) discretization methods, and their ratio.

The preceding results show that the proposed method exhibits high accuracy; however, its precision still needs to be further verified. Figure 18 presents the SD of TVD obtained from 10 repeated discretizations, using the same discretization conditions as in Figure 17, with the proposed method applied to different total grain counts. For comparison, the figure also shows the SD of TVD obtained from 10 repeated discretizations using the conventional method under a uniform GSD, with TVD estimation performed using nine equal-width bins. It can be seen that the SD of TVD for the conventional method is significantly larger than that for the proposed method.

To quantify the difference, the ratio between the SD of the conventional method and that of the proposed method is also calculated and depicted as a burnt orange curve in Figure 18. As the grain count increases, this ratio exhibits a trend of initial increase followed by a decline, with the inflection point occurring at approximately 500 grains. After the inflection point, the SD of the conventional method continues to decrease rapidly, while the SD of the proposed method tends to stabilize, which is likely the main reason for the observed inflection in the ratio.

Although the trend of the SD ratio is not monotonic, it remains at relatively high levels (approximately 100–700) within the grain-count range commonly encountered in engineering practice (10^2^–10^3^). In other words, the SD of TVD produced by the proposed method is about two orders of magnitude smaller than that of the conventional method, indicating a substantial improvement in precision. It should be noted that the conventional method applies only to uniform GSD, whereas the comparison here uses non-uniform GSD for the proposed method. Even so, the comparison remains informative, since applying the proposed method under uniform GSD would yield an SD approaching zero, and the discrepancy relative to the conventional method would only be larger. From a practical perspective, although grain ordering under non-uniform GSD does affect the TVD to some extent, the above results indicate that its influence remains limited within the grain-count range considered in this study. Therefore, seeking an optimal grain ordering is of limited practical significance, and repeated random shuffling is generally unnecessary to achieve acceptable discretization accuracy.

Taken together, the results demonstrate that the texture discretization method proposed in this work outperforms conventional methods in both accuracy and precision, enabling discretized textures with substantially higher fidelity.

## 6. Discussion

### 6.1. Influence of Randomness

**Figure 19:**
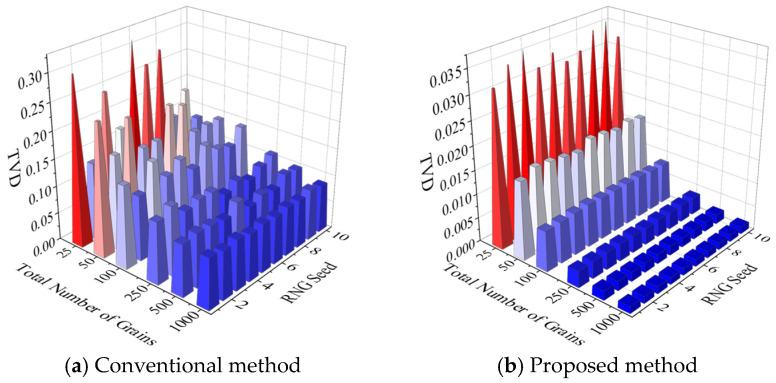
Comparison of TVDs from 10 repeated discretizations under the same settings as Figure 18.

Both the conventional texture discretization method and the method proposed in this study introduce stochastic processes, which impart a certain degree of randomness to the generated textures. Under fixed discretization conditions, both methods are capable of producing textures with random variability; however, such randomness inevitably leads to fluctuations in discretization error. The computed results reveal a substantial difference between the two methods in terms of the fluctuation magnitude of TVD, as illustrated in Figure 19. The figure compares TVD results obtained from ten repeated discretizations performed under identical conditions, where Figure 19a,b correspond to the conventional and the proposed methods, respectively. The randomness arising from the repeated discretizations in both methods is introduced solely through the use of different RNG seeds, and the discretization conditions and binning strategies are exactly the same as those used to obtain Figure 18.

**Figure 20:**
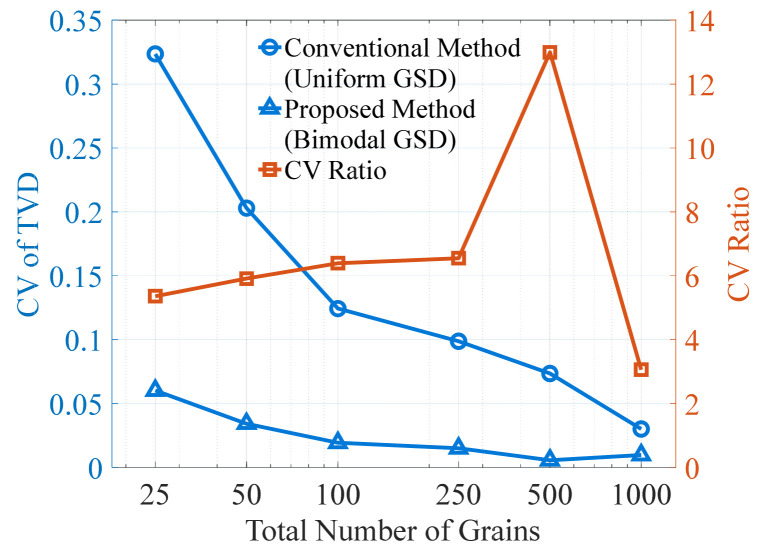
CVs of the TVD values (shown in Figure 19) from the conventional and proposed discretization methods, and their CV ratios.

It can be observed that TVD obtained by both methods exhibits pronounced fluctuations, and the fluctuation magnitude decreases as the total grain count increases. The key difference lies in the extent of these fluctuations: at the same grain count, the TVD variation produced by the proposed method is significantly smaller than that of the conventional method. For the proposed method, TVD fluctuations are clearly visible only when the total grain count is below 100, and become nearly indistinguishable beyond this value. To further analyze the dispersion of discretization error, Figure 20 presents the coefficient of variation (CV) of TVD for both methods. The results show that the CV decreases with increasing grain count for both methods, while the difference between them is gradually reduced. When the CVs of the two methods are divided, the resulting ratio shows a rise-then-fall trend, reaching a maximum of 13 at a total grain count of 500 and decreasing to a minimum of 3 at 1000 grains.

Although the fluctuations in discretization error arise from randomness in both methods, the difference in fluctuation magnitude originates from their distinct mechanisms of stochastic influence. The main difference lies in the target of randomness. In the conventional method, randomness is applied to the source samples used in inverse transform sampling. In the proposed method, in contrast, it is applied to the ordering of grains. In the conventional method, random errors in source samples are directly propagated into the discretized texture, making it difficult for the probability mass of each statistical bin to precisely match its target value. In contrast, the proposed method always satisfies the required probability mass of each bin, regardless of the grain arrangement. This fundamental difference enables the proposed method to achieve higher fidelity under identical discretization conditions, thereby underpinning its superiority over the conventional approach. This constitutes the core innovation of this study.

### 6.2. Influence of Periodicity

The periodicity of microstructural models is one important aspect in representing heterogeneous materials [51,52]. When a periodic model is combined with periodic boundary conditions (PBCs), the resulting simulations generally achieve more favorable accuracy and stability [53,54]. For microstructural models containing texture, periodicity manifests not only in the geometric arrangement of grains but also in the distribution of their crystallographic orientations. Specifically, the periodicity of texture is primarily reflected in the matching relationships among the orientations of grains located on opposite boundaries of the model. However, the potential impact of any inherent geometric periodicity of the model on the discretization of textures remains unclear and necessitates further investigation.

**Figure 21:**
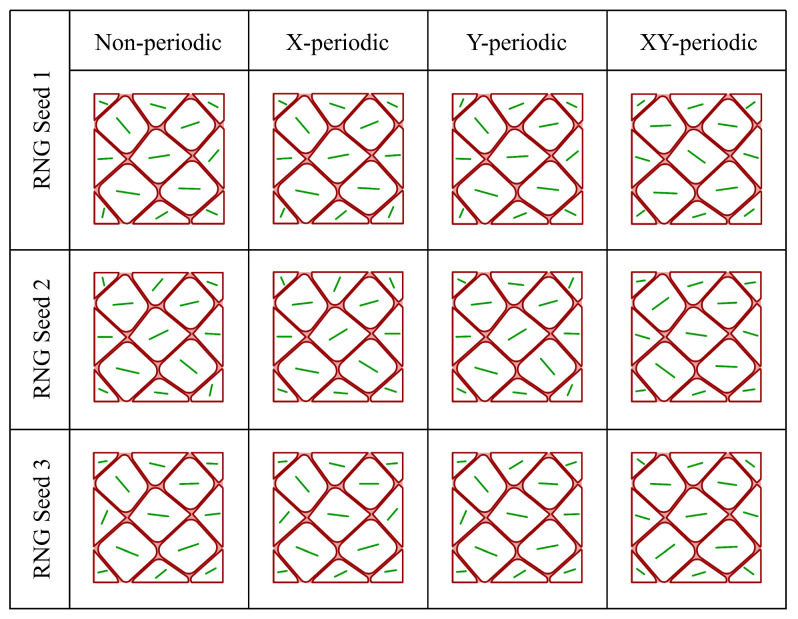
Discretized textures of the same microstructure under different PBCs using the proposed method (green lines indicate the orientations of individual grains).

For two-dimensional microstructural models, four types of boundary constraints are typically feasible: non-periodic, transverse periodic (X-periodic), longitudinal periodic (Y-periodic), and fully periodic (XY-periodic). Using the same geometric configuration, Figure 21 presents qualitative examples of the texture discretization results obtained by the proposed method under these four boundary conditions. All grains at the model boundaries are geometrically complementary, thereby providing the geometric basis for applying various PBCs. It can be observed that once the geometric boundary condition is specified, the resulting discretized texture remains consistent with the corresponding periodic characteristics. Repeated discretization with different random seeds consistently preserves this periodicity.

In the algorithmic workflow shown in Figure 3, the texture discretization procedure does not explicitly incorporate any operations associated with texture periodicity. Nevertheless, the discretized textures exhibit periodicity fully compatible with the imposed PBCs. This behavior can be understood from the fact that the discretization process is determined by grain-volume information, and the volume sequence inherently encodes its own periodic characteristics. Therefore, under the conditions considered in this work, the proposed method shows good compatibility with boundary-condition-induced periodicity: it does not require predefined periodicity parameters, the generated textures remain consistent with the periodicity implied by the boundary constraints, and the same framework can be applied to different boundary types ranging from non-periodic to fully periodic.

It should be noted that the present discussion is qualitative, since no dedicated quantitative metric for texture periodicity is introduced in this work. A quantitative characterization of texture periodicity and its more systematic evaluation will be considered in future work.

## 7. Conclusions

This study extends the conventional inverse transform sampling method by reconstructing the distribution of source samples used in the inverse transform, upon which a new texture discretization method is developed. Within the present framework, the method can accommodate different GSDs without introducing additional parameters beyond grain volumes. Theoretical derivations demonstrate that the resulting discrete texture strictly satisfies the original distribution in the probability mass across all orientation bins, thereby validating the correctness and robustness of the method.

For error evaluation, TVD is adopted as the quantitative metric, and the effects of three critical factors—grain ordering, total grain count, and binning strategy—are systematically examined. Comparative results across various GSDs indicate that the proposed method, relative to the conventional method, reduces the TVD by one order of magnitude and the standard deviation of the TVD by two orders of magnitude. This leads to a marked improvement in both the accuracy and precision of the discretized results.

To further elucidate the underlying mechanism of the method, the study also analyzes the randomness and periodicity characteristics of the discrete textures. The results indicate that the proposed method maintains high fidelity while also producing reasonable texture diversity. Moreover, it naturally conforms to the periodicity imposed by geometric constraints, resulting in more physically consistent discrete patterns.

Overall, the proposed method provides a concise, efficient, and highly adaptable approach for high-fidelity texture modeling, offering a robust statistical foundation for the representation of polycrystalline microstructures. This, in turn, may benefit downstream microstructural simulations and the associated mechanical predictions. A systematic investigation of how the reduced TVD achieved by the proposed method translates into improved microstructural simulation outputs will be an important topic of future work.

## Figures and Tables

**Figure A1:**
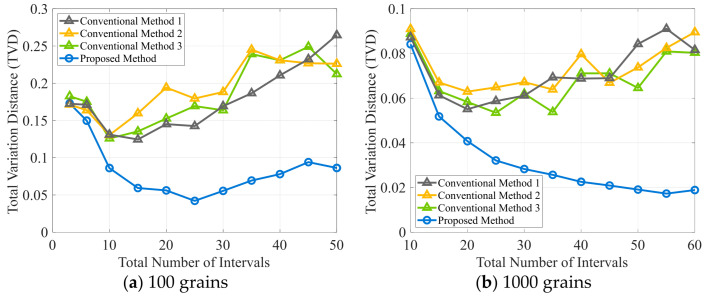
Effect of bin count on TVD obtained from various discretization methods and grain counts, using equal-interval binning.

## Data Availability

The original contributions presented in this study are included in the article. Further inquiries can be directed to the corresponding author.

## DURC Statement

Current research is limited to the field of computational materials science and numerical microstructure modeling, which is beneficial for developing robust algorithms for high-fidelity texture discretization and microstructure simulations. The work is strictly computational and theoretical, involving orientation distribution functions and grain volume discretization, and does not include any experimental handling, synthesis, or manipulation of explosives or other hazardous substances. It therefore poses no threat to public health or national security. The authors acknowledge the dual-use potential of the research in computational methods that could theoretically be applied to various engineering materials, and confirm that all necessary precautions have been taken to prevent potential misuse. The authors strictly comply with national and international DURC regulations and advocate for responsible application, ethical conduct, regulatory compliance, and transparent reporting to ensure that the outcomes remain purely beneficial.

## Abbreviations

The following abbreviations are used in this manuscript:

ODF	Orientation Distribution Function
CDF	Cumulative Distribution Function
GSD	Grain Size Distribution
TVD	Total Variation Distance
LVD	Local Variation Distance
PBX	Polymer-Bonded Explosive
RNG	Random Number Generator
GMM	Gaussian Mixture Model
SD	Standard Deviation
CV	Coefficient of Variation
PBC	Periodic Boundary Condition

## Appendix A. Effect of Bin Count on TVD Estimates with Equal-Interval Binning

When equal-interval binning is used, the number of bins has a significant influence on the estimated TVD, as illustrated in Figure A1. The figure presents the relationship between the bin count and the resulting TVD, comparing the conventional inverse transform sampling method with the proposed discretization method. All discretizations employ uniform GSDs, and two cases with total grain counts of 100 and 1000 are considered. For the conventional method, three discretizations are performed using different RNG seeds.

The results indicate that the dependence of TVD on the number of bins is non-monotonic, showing an underlying trend of an initial decrease followed by an increase. Both the conventional and proposed methods exhibit a critical number of bins that minimizes the TVD. However, determining this critical value is not straightforward—particularly for the conventional method, whose TVD values display substantial variability across repetitions. Furthermore, the critical bin count depends on the total number of grains: when the grain count is 100, the conventional method yields a critical value of approximately 10 bins; when the grain count increases to 1000, this value rises to about 20.

Accordingly, in the comparison between the conventional and proposed methods (Section 4.4) and in the validation of the proposed method (Section 5.2), appropriate bin counts are chosen respectively to ensure meaningful and reliable error assessments.
